# Targeting Attenuated Interferon-α to Myeloma Cells with a CD38 Antibody Induces Potent Tumor Regression with Reduced Off-Target Activity

**DOI:** 10.1371/journal.pone.0162472

**Published:** 2016-09-09

**Authors:** Sarah L. Pogue, Tetsuya Taura, Mingying Bi, Yong Yun, Angela Sho, Glen Mikesell, Collette Behrens, Maya Sokolovsky, Hussein Hallak, Moti Rosenstock, Eric Sanchez, Haiming Chen, James Berenson, Anthony Doyle, Steffen Nock, David S. Wilson

**Affiliations:** 1 Teva Pharmaceuticals, Global Branded Biologics Division, Redwood City, California, United States of America; 2 Teva Pharmaceuticals, Global Branded Biologics Division, Sydney, Australia; 3 Teva Pharmaceuticals, Global Branded Biologics Division, Netanya, Israel; 4 The Institute for Myeloma and Bone Cancer Research, West Hollywood, California, United States of America; University of Oxford, UNITED KINGDOM

## Abstract

Interferon-α (IFNα) has been prescribed to effectively treat multiple myeloma (MM) and other malignancies for decades. Its use has waned in recent years, however, due to significant toxicity and a narrow therapeutic index (TI). We sought to improve IFNα’s TI by, first, attaching it to an anti-CD38 antibody, thereby directly targeting it to MM cells, and, second, by introducing an attenuating mutation into the IFNα portion of the fusion protein rendering it relatively inactive on normal, CD38 negative cells. This anti-CD38-IFNα(attenuated) immunocytokine, or CD38-Attenukine™, exhibits 10,000-fold increased specificity for CD38 positive cells in vitro compared to native IFNα and, significantly, is ~6,000-fold less toxic to normal bone marrow cells in vitro than native IFNα. Moreover, the attenuating mutation significantly decreases IFNα biomarker activity in cynomolgus macaques indicating that this approach may yield a better safety profile in humans than native IFNα or a non-attenuated IFNα immunocytokine. In human xenograft MM tumor models, anti-CD38-IFNα(attenuated) exerts potent anti-tumor activity in mice, inducing complete tumor regression in most cases. Furthermore, anti-CD38-IFNα(attenuated) is more efficacious than standard MM treatments (lenalidomide, bortezomib, dexamethasone) and exhibits strong synergy with lenalidomide and with bortezomib in xenograft models. Our findings suggest that tumor-targeted attenuated cytokines such as IFNα can promote robust tumor killing while minimizing systemic toxicity.

## Introduction

Multiple myeloma (MM) is the second most common blood cell malignancy in the U.S. after non-Hodgkin’s lymphoma [1, 2]. Current treatments for MM include chemotherapy, steroids, immunomodulatory drugs, proteasome inhibitors and stem cell transplantation. Despite the increased efficacy of these therapies, nearly all patients eventually relapse and become refractory to treatment [3]. Thus, MM remains an incurable disease with a 47% five-year survival rate [1, 3, 4].

IFNα is a pleiotropic proinflammatory cytokine with demonstrated anti-proliferative, cytotoxic and anti-neoplastic immunomodulatory activity [5, 6]. It has been used for decades to treat viral infections and certain cancers including MM [7]. While initial trials testing IFNα as maintenance therapy for MM yielded inconsistent results, subsequent meta-analyses showed significant improvement in survival rates, although tolerability was poor [8]. The range of serious side effects frequently associated with IFNα include nausea, severe flu-like symptoms, vasculopathic complications (e.g., decreased leucocytes and platelets), and sometimes depression or anxiety [9–12]. In one MM study, maintenance therapy with IFNα was discontinued in up to 37% of patients in due to toxicity [13]. Such widespread toxicity coupled with the typically high doses of IFNα required for efficacy in MM patients translates into a narrow therapeutic index (TI) for IFNα, defined as the ratio between maximum tolerated dose and minimum therapeutic dose. The narrow TI of IFNα has limited its consistent clinical use for the treatment of MM.

One approach to decrease the marked toxicity of cytokines in general in cancer therapy is to attach them to tumor-targeting antibodies or antibody fragments. This promotes increased local concentration of the cytokines at tumor sites [14, 15]. Such “immunocytokines” have been described extensively, including those based on IFNα [16–24]. While potentially decreasing the effective dose, this strategy does not address, and may compound, the issue of IFNα toxicity due to the extended half-life generally observed with antibody based therapies and the ubiquitous expression of the interferon-α receptor (IFNAR) on non-tumor cells.

Here, we describe our approach to broaden the TI of IFNα by minimizing its systemic toxicity while retaining its potent anti-tumor activity. We chose the MM tumor antigen CD38 as our target antigen because it is expressed at high levels on nearly all MM tumor cells and has limited normal tissue expression [25–27]. We engineered a mutation into the IFNα portion of the CD38-targeted immunocytokine to significantly reduce its binding to IFNAR on CD38-negative cells. Our data shows that this CD38-targeted, attenuated IFNα immunocytokine, dubbed “CD38-Attenukine™”, is orders of magnitude less potent at stimulating antigen-negative cells than native IFNα, and yet maintains potent anti-tumor activity on antigen-positive cells. In most cases, treatment with CD38-targeted IFNα attenuated Attenukine™ leads to complete elimination of even very large, established human MM tumors in mice.

## Materials and Methods

### IFNα constructs and fusion proteins

Reference anti-CD38 antibody variable regions were generated by PCR from published V region sequences (reference antibody [28] as described in WO 2013/059885). Negative control, non-targeted irrelevant specificity, V-region sequences (anti-yellow fever virus clone 2D12 [29]) were generated from published sequences (WO 2013/059885). Negative control sequences (anti-respiratory syncytial virus) used in the cynomolgus study were generated from published sequences (WO 2013/059885). The human IFNα2b gene was isolated from HEK293 genomic DNA by standard PCR methods using primers (5’-GGTAAATCCGGAGGCGGCGGGAGCTGTGATCTGCCTCAAACCCACAGCCTG-3’ and 5’ACGTGGATCCTATTCCTTACTTCTTAAACTTTCTTGC-3’). Attenuating mutations in IFNα2b were introduced by PCR at residues which interact with the high affinity IFNα receptor chain, IFNAR2 [30]. Anti-CD38 or irrelevant V-region and IFNα2b gene fragments were cloned into the pTT5 mammalian expression vector [31] containing human IgG4 and kappa immunoglobulin constant region genes. Naked antibody and antibody-IFNα fusion proteins were transiently expressed in HEK293-6E cells [31] and purified using Protein G-Sepharose columns (GE Healthcare, Piscataway, NJ). ELISA binding assay used to determine relative affinities of the wild type and mutated IFNα fusion proteins for the IFNAR2. Specifically, 96-well plates were coated with 5 μg/ml human IFNAR 2-human Fc fusion protein (R & D Systems, Minneapolis, MN). After brief washing with PBS, wells were blocked with Superblock blocking solution (ThermoFisher Scientific, Waltham, MA) and loaded with anti-CD38-IFNα fusion proteins diluted in PBS with 0.05% Tween 20 (PBST). After 60 min incubation at room temperature, wells were washed with PBST, and anti-human κ-horseradish peroxidase conjugate (SouthernBiotech, Birmingham, AL) diluted 1/5000 in PBST was applied to each well to capture IFNAR2-anti-CD38-IFNα fusion complexes. Following 60 minutes incubation and washing with PBST, 3,3’,5,5’-tetramethylbenzidine (TMB, Sigma Aldrich, St. Louis, MO) was added to wells to develop the colorimetric signal which was measured by a vmax plate reader after color development was stopped with sulfuric acid.

### Bone marrow colony forming assays

Frozen bone marrow (BM) mononuclear cells from MM patients (AllCells, Inc., Alameda, CA) and MM cell lines (American Type Culture Collection, ATCC) were cultured in MethoCult H4230 media (Stem Cell Technologies, Vancouver, Canada) with 10% FBS and 10% phytohemagglutin (PHA) stimulated leukocyte conditioned medium. Normal BM cells (AllCells, Inc.) were cultured in MethoCult H4434 media. All BM cells were incubated with either vehicle (PBS) or 10,000 IU/ml IFNα Intron® A (Schering Corp. Merck, NJ) at 37°C in 5% CO2 for 7 or 14 days before colonies were stained with SYBR green and visually counted using a microscope. Bone Marrow cells: Normal and myeloma bone marrow cells were purchased from AllCells, Inc. as frozen vials containing ten million bone marrow mononuclear cells/vial. All donors signed an informed consent release and were made aware that the cells were to be used for research purposes. Donors were all over the age of 18.

### MM cell viability and IFNα activity assays

Viability of MM cell lines cultured with and without 10,000 IU/ml IFNα2b (US Biological, Salem, OR) was determined using the CellTiter-Glo® luminescent assay (Promega, Madison, WI). MM cell lines included ARH-77 (ATCC® CCL-155™), RPMI8226 (ATCC® CRL-1621™), NCI-H929 (ATCC® CRL-9068™), U266 (ATCC® TIB-196™) and ARP-1 (Myeloma Institute, University of Arkansas, Little Rock, AK). IFNα activity on CD38-negative reporter cells (iLite™ cell line) was determined using the iLite™ Cell Assay following a modified manufacturer’s protocol (PBL Assay Science, Piscataway, NJ).

### Flow cytometry

BM cells from healthy and MM patients (AllCells, Inc.) were blocked with human IgG, washed with PBS and stained with 50 μg/ml naked CD38 or CD138 antibodies (eBiosciences, San Diego, CA). Cells were then incubated with goat anti-mouse IgG, F(ab’)2-PE for 20 minutes on ice and washed twice. FACS acquisition was done on a BD FacsCalibur™ using CellQuest™ software.

### Xenograft Studies

All mouse experiments received approval from the Charles River Labs and Los Angeles Biomedical Research Institute Animal Care Committees and were conducted according to the Institutional Animal Care and Use Committee and in adherence to the National Institutes of Health “*Guide for the Care and Use of Laboratory Animals”* [32] at Charles River Labs and at Los Angeles Biomedical Research Institute at Harbor-UCLA, both AAALAC accredited institutions. Any animal exhibiting weight loss, lethargy, hunched posture, or ruffled fur that did not improve after treatment holiday was euthanized. When tumor volume reached endpoint, or at the end of study, animals were euthanized by the use of CO2 for rodent euthanasia per institutional protocols. In subcutaneous models, mice were euthanized when tumor volume endpoint was reached. In these studies, no mice exhibited weight loss, lethargy, hunched posture or ruffled fur. In the systemic myeloma model, MM1S, 30 of 40 mice (all control groups), were euthanized due to weight loss, lethargy and ruffled fur (symptoms of disease, study endpoint) prior to end of study.

### Non-human Primate study

#### Source

The cynomolgus monkeys (Macaca fasicularis) used in this study were of Chinese origin and were received from Primus Bio-Resources Inc., 531, boulevard des Pairies, Bldg 25, Laval, Quebec, Canada, H7V 1B7 on April 10, 2013. A total of 23 animals (3–4 animals per test group) were treated and evaluated.

#### Animal Care Committee

The non-human primate study was performed by AAALAC accredited ITR Laboratories Canada, Inc., Baie D'Urfé, Québec, Canada. The study plan was reviewed and assessed by the Animal Care Committee (ACC) of ITR. ACC acceptance of the study plan was maintained on file at ITR. All animals used on this study were cared for in accordance with the principles outlined in the current "*Guide to the Care and Use of Experimental Animals*" as published by the Canadian Council on Animal Care [33] and the "*Guide for the Care and Use of Laboratory Animals*", a NIH publication [32]. The study did not unnecessarily duplicate previous experiments.

#### Health Status

On arrival at ITR, monkeys were weighed and then subjected to a detailed physical examination to ensure satisfactory health status. In addition, stool samples were collected and examined for the presence of fecal parasites using the fecal flotation method [34]. During the quarantine period, all animals were tested twice for tuberculosis by intradermal injection of tuberculin.

#### Housing

Each monkey was housed in a stainless steel cage equipped with an automatic watering system. Each cage was labeled with a color-coded cage card indicating the study, group and animal numbers, sex and dose level. Animals were housed a total of 15 days individually. Cages were all in the same room providing animals with visual, olfactory and auditory stimulation/access to one another.

#### Room Environment

The animal room environment was controlled (targeted ranges: temperature 21 ± 3°C, relative humidity 50 ± 20%, 12 hours light, 12 hours dark [except during designated procedures] and a minimum of 10 air changes per hour). Temperature and relative humidity were monitored continuously and records are maintained at ITR.

#### Diet/Water

A standard certified commercial primate chow (Teklad Certified Primate Chow #2055C) was available ad libitum to each monkey except during designated procedures. Municipal tap water (which was purified by reverse osmosis, ultraviolet light and further filtered with a 0.2 μm filter) was provided to the animals ad libitum except during designated procedures.

#### Environmental Enrichment

Animals were offered certified treats, non-certified treats (e.g., frozen yogurt, banana, or grapes; nuts, peanuts, raisins) and other non-dietary items (e.g., toys) as part of the ITR environmental enrichment program at appropriate intervals. More specifically, for appetite enhancement, fruit, vegetables or certified/non-certified treats were offered to each monkey once daily during all phases of the study.

#### Acclimation

An acclimation period of approximately 4 weeks (approximately 3 weeks of quarantine and 1 week of pretreatment procedures) was allowed between receipt of the animals and the start of dosing to accustom the monkeys to the laboratory environment. Monkeys were acclimated to the experimental procedures (e.g., sling restraint for intravenous infusion) for 3 consecutive days prior to the start of dosing.

#### Administration of the Test Items

Each test item dose formulation was administered once by intravenous infusion over a period of 1 hour via a disposable indwelling catheter inserted into one of the saphenous veins.

#### Monitoring

All monkeys were monitored daily for mortality, or clinical signs involving general appearance, posture, gait, respiration, behavioral and coordination abnormalities, and abnormal feces. More careful examination of an individual animal’s body, mouth, nose, ears, eyes, skin, and coat were included if any initial abnormalities were noted. Body weights were monitored weekly as well as on days -2, 1 and 8. No animals became ill or injured at any point during the study.

#### Sampling

A series of 10 blood samples (approximately 0.5 mL each for time point up to 48 h post end of infusion and approximately 1 mL each for 96 h to 168 h post end of infusion) was collected from each monkey at the following time points: Pre-dose, 0 minutes (immediately post end of infusion), 2, 6, 12, 24, 48, 96, 120 and 168 hours post end of infusion. Serum samples were evaluated for biomarker analysis.

#### Terminal Procedure

Following collection of the last blood samples on Day 8, all animals were released to the ITR spare colony.

### Xenograph tumor models

LAGκ-1A, LAGκ-2, and LAGλ1 tumor fragments (20–40 mm^3^), obtained from in vivo passaged MM patient cells from 3 donors, were implanted intramuscularly into CB.17 SCID mice as described [35, 36]. Treatment started on day 8 following implantation. Tumor volumes, M protein and mouse body weights were measured twice weekly by caliper; endpoint was reached at tumor volume of 2000 mm^3^. The sources of these tumor cells were: LAGk-2, date of collection: 11/9/06, 68 year old male, diagnosed with myeloma, bone marrow aspirate obtained from patient by informed consent, LAGk-1A, date of collection 9/15/04, 76 year old female, diagnosed with myeloma, bone marrow aspirate obtained by informed consent, LAGλ-1, date of collection >10 years ago, female diagnosed with myeloma, tumor cells obtained by informed consent. Cell line models: 10 million NCI-H929 (ATCC® CRL-9068™) or Daudi cells (ATCC® CCL-213™) were mixed 1:1 with Matrigel and injected subcutaneously into the hind flank. Tumor volumes and mouse body weights were measured twice weekly by caliper. As a direct measurement of tumor growth, calipers were used to assess tumor volume twice weekly, and the formula for an ellipsoid volume was applied 4/3π x [width/2]^2^ x [length/2]. Endpoint was reached at tumor volume of 2000 mm^3^. For the systemic multiple myeloma model, MM1S 10 million cells (ATCC® CRL-2974™) were injected intravenously into the tail vein; mouse body weight and overall health were monitored twice weekly with a survival endpoint.

### Statistical Analysis

For all in vitro experiments, means of two or three samples +/- Standard Deviation are presented. For in vivo studies, tumor volumes are largely represented at mean tumor volume of 10 mice +/- Standard Error of Mean. P values were calculated using Students t-Test.

## Results

### IFNα inhibits growth of human MM cells

**Fig 1:**
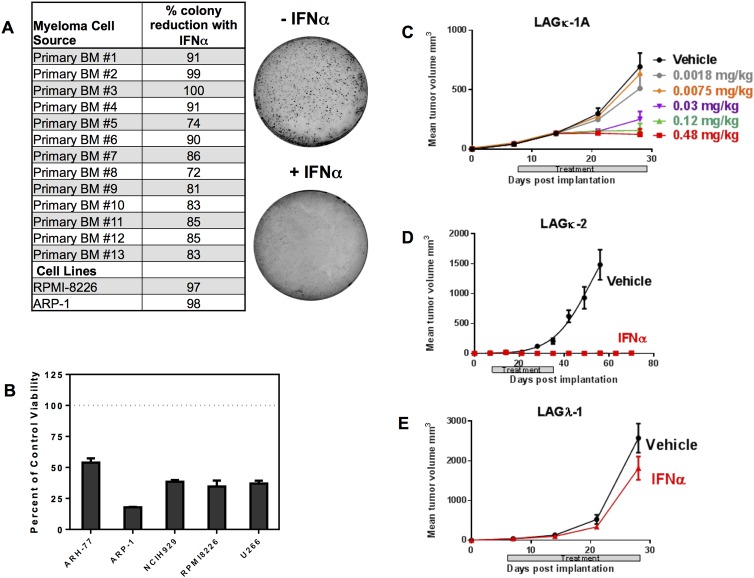
High IFNα exposure inhibits human MM tumor growth in vitro and in vivo. (A) Bone marrow (BM) cells from 13 MM patients and 2 human MM cell lines were grown in colony supporting matrix with or without IFNα (10,000 IU/ml). Percent reduction in colony number from each donor was calculated relative to untreated control wells. Representative images for one primary MM bone marrow sample (#6) are shown with and without IFNα treatment. (B) Five MM cell lines were cultured for 3 days in standard cell media with or without 10,000 IU/ml IFNα. Percent viability of each cell line relative to untreated cells (dotted line) is shown. Data represent means from triplicate wells +/- SD. (C, D, E) IFNα treatment of 6–8 week old CB17 SCID mice implanted with mouse-passaged human MM tumor fragments (LAGκ-1A, LAGκ-2, and LAGλ-1). On day 8 post-tumor implantation, recipient mice (n = 10 per group) were treated with IFNα or vehicle twice a week for 4 weeks. The treatment windows for all experiments are indicated by shaded bars. Mean tumor volumes +/- SEM are shown. (C) In a dose-response study, only high doses of IFNα (0.12mg/kg and 0.48 mg/kg) were effective at completely inhibiting LAGκ-1A tumor growth. (D) Treatment with IFNα 0.48 mg/kg) also strongly inhibited LAGκ-2 tumor growth. On Day 56, p = <0.0001. (E) Treatment with IFNα (0.24 mg/kg) did not significantly inhibit LAGλ-1 tumors (p = 0.7537), which originated from a bortezomib-refractory MM patient. All statistical analyses were performed using Student’s t-Test.

IFNα is known to inhibit tumor growth of numerous types of cancers including lymphomas and leukemias [37]. To evaluate the effects of IFNα on human MM cells in vitro, we assessed its activity in colony forming assays using BM samples from MM patients and MM cell lines. We found that IFNα strongly inhibited the growth of all tested MM specimens (Fig 1A and 1B). When grown in the presence of 10,000 IU/ml of IFNα colony counts from primary MM cells and MM cell lines were reduced by 72–100% compared to untreated controls (Fig 1A). In cell culture proliferation assays, IFNα treatment reduced proliferation between 50 to 85% compared to controls (Fig 1B).

To evaluate the activity of IFNα on primary MM tumors in vivo, we performed xenograft models using in vivo passaged MM tumor fragments [35, 36]. Fragments of tumors LAGκ-1A, LAGκ-2 and LAGλ-1 were implanted intramuscularly into SCID mice on day 0, and IFNα or vehicle treatments were initiated on day 8. In an IFNα dose response study using the LAGκ-1A model, human IFNα at doses of 0.12–0.48 mg/kg strongly inhibited growth of tumors in all (10/10) mice (Fig 1C). Similarly, growth of LAGκ-2 tumors was also strongly inhibited by high dose (0.48 mg/kg) IFNα treatment (Fig 1D). All mice (10/10) were tumor free within two weeks of treatment and remained so following cessation of treatment. In contrast, LAGλ-1 tumors, originating from a bortezomib-refractory MM patient, did not show a significant reduction in tumor growth even with high dose (0.24 mg/kg) IFNα treatment (Fig 1E). We note that the 0.12 to 0.48 mg/kg doses of IFNα are orders of magnitude higher than typical IFNα doses given to cancer patients (approximately 1μg/kg). Such high IFNα doses are tolerated in animal experiments without toxicity because human IFNα does not efficiently stimulate murine IFNAR [38, 39]. These experiments reveal the potential of IFNα to exert profound direct anti-MM tumor activity in vivo if sufficient exposure is achieved.

### Antibody-IFNα fusion protein displays high specificity for CD38 positive cells

**Fig 2:**
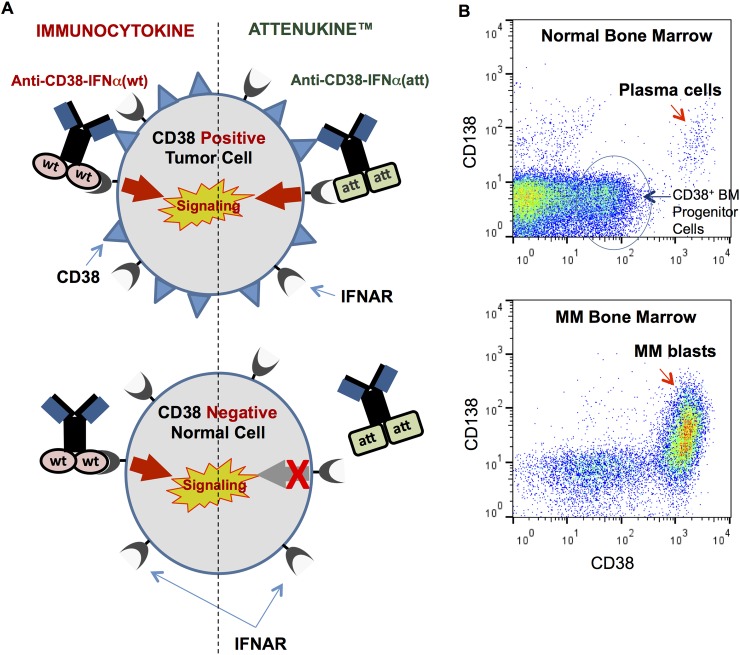
Experimental strategy to enhance the therapeutic index of IFNα in MM treatment. (A) Experimental strategy: Left of dotted line, the standard immunocytokine approach targeting CD38 positive tumor cells (upper scheme) directs wild type IFNα to the tumor via an anti-CD38 antibody, causing IFNα signaling through IFNAR. Because IFNα has such high affinity for its receptor, signaling also occurs in CD38 negative (normal) cells (lower scheme) leading to systemic toxicity that is typically associated with IFNα treatments. With the Attenukine™ approach (right side of dotted line), the IFNα portion of the molecule is mutated to significantly reduce binding to its receptor. Despite the attenuation, anti-CD38-IFNα(attenuated) maintains potent activity on CD38 positive MM cells which is mediated by the strong antibody-antigen interaction on tumor cells (upper scheme), with little to no effect on CD38 negative normal cells (lower scheme). Thus, the Attenukine™ approach reduces toxicity on normal cells while maintaining high potency on antigen positive tumor cells. (B) A FACS plot of normal BM (upper plot) shows that high CD38 expression is restricted to plasma cells, with low levels of CD38 expressed on a subset of CD138 negative progenitor cells and lymphocytes. The FACS plot of MM BM (lower plot) shows that MM blasts express high levels of CD38.

Despite the strong potential of IFNα to treat MM, its side effects limit clinical use [9–12]. In an effort to broaden the TI of IFNα, we first employed a conventional immunocytokine approach. We produced a fusion protein, designated anti-CD38-IFNα(wt), consisting of wild-type human IFNα2b fused directly to the C-terminus of a human IgG4 anti-CD38 heavy chain (Fig 2A). CD38 is highly expressed on nearly all MM tumor cells and normal human plasma cells, with low expression on other normal BM precursor subsets (Fig 2B) [40].

**Fig 3:**
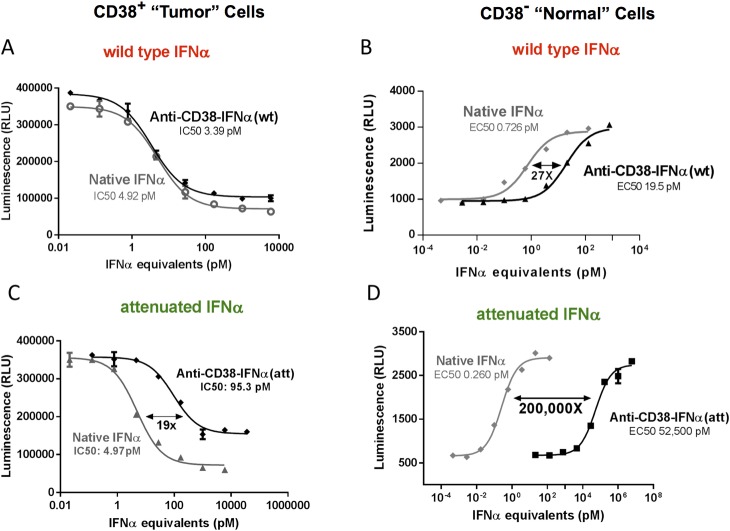
Anti-CD38-IFNα(att) retains strong on-target tumor cell activity with reduced off-target activity in vitro. Two assays were used to evaluate the antigen specificity of anti-CD38 targeted IFNα’s. The graphs on the left show viability assays of treated ARP-1 MM cells three days after incubation with IFNα compounds. This assay predicts on-target, anti-tumor activity of IFNα and IFNα-fusion proteins. The graphs on the right show IFNα responses of CD38-negative reporter cells that express a reporter gene driven by the IFNα response element (IRE). This response predicts IFNα activity on CD38-negative, normal cells. Both the cell viability and IRE activity are measured by luminescence and presented as relative luminescence units (RLU). Data represent means from triplicate measurements +/- SD. (A) Activity of the wild type IFNα immunocytokine, anti-CD38-IFNα(wt) (black diamonds), has similar potency as native IFNα (gray circles) on CD38-positive MM ARP1 cell viability. (B) The IRE activity of the wild type IFNα immunocytokine is 27-fold less potent than native IFNα on the CD38-negative reporter cell line. (C) The activity of the attenuated IFNα fusion protein, anti-CD38-IFNα(att), is 19x less potent than native IFNα on the CD38 expressing ARP-1 cell line. (D) The activity of anti-CD38-IFNα(att) is 200,000x weaker than native IFNα on the CD38-negative reporter cell line.

When evaluated in vitro, the anti-CD38-IFNα(wt) immunocytokine proved highly effective at inhibiting CD38 positive ARP1 MM cell proliferation, with a similar potency as that of unmodified, wild type IFNα (hereafter referred to as native IFNα) (IC50s of 3.39 pM vs 4.92 pM, respectively; Fig 3A). Given the high affinity of IFNα for its receptor, this fusion protein also stimulated potent IFNα activity on CD38 negative cells (Fig 3B) although with measurably reduced activity compared to native IFNα (19.5 pM vs 0.726 pM, respectively). This reduced activity is likely due to steric constraints created by fusion of IFNα to the antibody.

To eliminate the IFNα activity on normal cells, we engineered various point mutations into the IFNα portion of the fusion protein to reduce its affinity for IFNAR. We hypothesized that normal cells which express low or no CD38 would be relatively unresponsive to the attenuated IFNα (Fig 2A, lower diagram). The CD38 antibody portion of the immunocytokine would, however, direct a high local concentration of attenuated IFNα to the surface of CD38 positive tumor cells (Fig 2A, upper diagram), thereby compensating for the weakened binding of IFNα to IFNAR and restoring activity on the targeted tumor cells.

To identify the optimal IFNα mutation, we constructed a large panel of anti-CD38-IFNα fusion proteins (Attenukines™) with specific IFNα mutations at residues which interact with the high affinity IFNα receptor chain, IFNAR2 [30]. Representative constructs and their relative binding affinities to IFNAR2 are shown in Table 1. Each construct was evaluated in vitro for potency in the CD38 positive and CD38 negative cell line assays. One Attenukine™, designated anti-CD38-IFNα(A145D), hereafter referred to as anti-CD38-IFNα(att), was selected for further study based on optimal in vitro on-target and off-target cell activity and high production levels. Anti-CD38-IFNα(att) strongly inhibited MM tumor cell proliferation, although it displayed approximately 20x lower potency relative to native IFNα(IC50s of 95.3 pM and 4.97 pM, respectively; Fig 3C). More notably, on CD38-negative cells, the anti-CD38-IFNα(att) Attenukine™ was 200,000-fold less potent than native IFNα(EC50's of 52,200 pM and 0.260 pM, respectively) (Fig 3D). Thus, anti-CD38-IFNα(att) exhibited negligible IFNα activity on normal, CD38 negative cells while retaining strong anti-tumor activity on CD38 positive tumor cells.

**Table 1:** IFNα attenuating mutations in CD38- Attenukines™ and relative binding affinities to IFNAR2.

Construct	Codon and Position		Relative Binding Affinity
	**144**	**145**	
**αCD38-IFNα(wt)**	AGA	GCA	1.0
**αCD38-IFNα(A145G)**	AGA	***GGA***	11.4
**αCD38-IFNα(R144A)**	***GCA***	GCA	18.6
**αCD38-IFNα(A145H)**	AGA	***CAC***	34.7
αCD38-IFNα(A145D)*	AGA	***GAT***	55.3
**αCD38-IFNα(R144T)**	***ACC***	GCA	91.6
**αCD38-IFNα(R144I)**	***ATC***	GCA	128.0

***Italic*** text indicates mutated codons.

The 144 and 145 residues within the IFNα molecule are among those which interact with the high affinity IFNα receptor chain, IFNAR2.[30]

*The αCD38-IFNα(A145D) construct is designated as anti-CD38-IFNα(att) in text.

To estimate the potential TI increase of anti-CD38-IFNα(att) and other IFNα Attenukines™ in vivo, we calculated an in vitro “antigen specificity index (ASI)” for each construct (Table 2). ASI is defined as the relative potency of an Attenukine™ on antigen-positive cells versus antigen-negative cells compared to native IFNα. An ASI of 1 indicates no antigen specificity and is the value assigned to native IFNα. An ASI of 100 indicates a 100-fold increased potency on antigen-positive cells (tumor) compared to antigen-negative cells (normal) relative to native IFNα. As shown in Table 2, the ASI for the Attenukine™ anti-CD38-IFNα(att) is 10,100, representing approximately 10,000-fold increased specificity compared to native IFNα for activity on CD38 positive versus CD38 negative cells. In contrast, the ASI for the targeted immunocytokine with native IFNα sequence [anti-CD38-IFNα(wt)], such as those recently described by others [16–24], is a mere 39. These in vitro findings predict a markedly broadened TI for the Attenukine™ anti-CD38-IFNα(att) compared to a non-attenuated IFNα immunocytokine.

**Table 2:** Antigen specificity index (ASI) of anti-CD38-IFNα and attenuated variants.

	Construct	CD38 Positive Cells		CD38 Negative Cells		
			(A)		(B)	(A/B)
		IC50 (pM)	IC50 IFN/IC50 variant	EC50 (pM)	EC50 IFN/EC50 variant	Antigen Specificity Index
**Exp I**						
	**Native IFNα**	4.92	1.00	0.726	1.00	1
	**αCD38-IFNα(wt)**	3.39	1.45	19.47	3.7x 10^−2^	39
**Exp II**						
	**Native IFNα**	4.97	1.00	0.26	1.00	1
	αCD38-IFNα(A145D)*	95.30	0.05	52 500	4.94x 10^−6^	10 115
	**αCD38-IFNα(A145G)**	15.30	0.25	2 040	1.27x 10^−4^	1 962
	**αCD38-IFNα(A145H)**	41.06	0.09	24 900	1.04x 10^−5^	8 619
	**αCD38-IFNα(R144A)**	138.30	0.03	25 800	1.01x 10^−5^	2 977
	**αCD38-IFNα(R144I)**	136.10	0.03	159 000	1.64x 10^−6^	18 346
	**αCD38-IFNα(R144T)**	281.20	0.01	30 800	8.44x 10^−6^	1 185

The values for native IFNα serve as internal reference points within each experiment and as controls for inter-experimental variability between Experiment 1 and Experiment 2.

*The αCD38-IFNα(A145D) construct is designated as anti-CD38-IFNα(att) in text.

### Reduced activity of anti-CD38-IFNα(att) on normal cells

**Fig 4:**
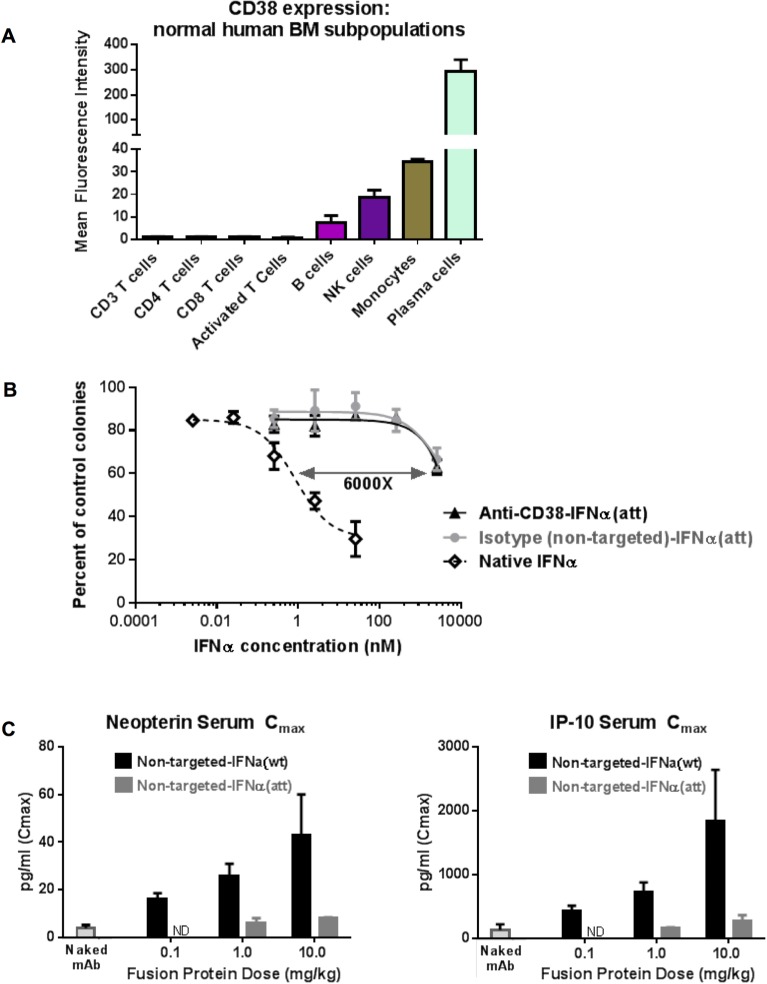
Attenuated IFNα fusion proteins have minimal activity on normal cells in vitro and in vivo. (A) CD38 expression on subsets of normal bone marrow cells as measured by FACS. Data represent means of two donor samples +/- SD. (B) Inhibition of normal bone marrow colony formation by anti-CD38-IFNα(att) (black solid line) or non-targeted-IFNα(att) (gray solid line) compared to native IFNα (black dashed line). The graph shows percentage of colony counts relative to untreated, control BM cells. Data represent means of three donor samples +/- SD. (C) Serum levels of IFNα induced biomarkers neopterin and IP-10 in cynomolgus monkeys (n = 3 or 4 per group) after a single dose of non-targeted, attenuated IFNα fusion protein (non-targeted-IFNα(att)), non-targeted wild type IFNα fusion protein (non-targeted-IFNα(wt)) or 10mg/kg naked non-targeted control IgG4 antibody. ND = Not Done. Data represent means from three or four monkey samples +/- SD.

The subsets of hematopoietic cells in normal BM and peripheral blood that express CD38 (Fig 2B) represent the normal cells most likely to be adversely affected by CD38-targeted therapies. We found that CD38 expression on peripheral and BM lymphocytes is approximately 100-fold less than that of plasma cells and myeloma blasts (Figs 2B and 4A). Thus, IFNAR stimulation of lymphocytes in patients by a CD38 targeted attenuated cytokine would be predicted to be relatively low compared to MM cells. Nevertheless, to evaluate in vitro whether anti-CD38-IFNα(att) mediates a toxic response in normal BM, we compared the activity of anti-CD38-IFNα(att) versus native IFNα in hematopoietic colony formation assays using cells isolated from healthy human BM donors. We also included an human IgG4 negative control fusion protein, designated “non-targeted-IFNα(att)”, that had irrelevant (non-CD38 binding) variable domains.

The results of these experiments showed that, as expected, native IFNα significantly inhibited BM colony formation, yielding, at the highest concentration tested (25 nM), only 25% of colonies formed compared to untreated cells (Fig 4B). In contrast, BM cultures treated with the same or 10X higher concentrations of either anti-CD38-IFNα(att) or a non-targeted-IFNα(att) fusion protein maintained good colony formation, yielding approximately 85% of the number of colonies formed compared to untreated cells. At a much higher concentration (6000 nM), colony formation dropped to 60% of control cultures for both molecules, corresponding to the inhibitory effect induced by approximately 1nM native IFNα. This indicates that attenuated IFNα (either targeted to CD38 or not) had approximately 6,000x less anti-proliferative activity than native IFNα on normal BM cells. Based on these findings, we would predict that the low level of CD38 expression on normal BM cells and peripheral lymphocytes is not sufficient to allow anti-CD38-IFNα(att) stimulation through the IFNα receptor at relevant drug concentrations and that BM toxicity in vivo may therefore be largely avoided.

To assess decreased off-target activity of the attenuated IFNα antibody fusion in vivo, we tested the activity of non-targeted-IFNα(att) versus non-targeted-IFNα(wt) in cynomolgus monkeys which, unlike mice, exhibit relevant and measurable responses to native human IFNα [41]. This study addressed whether normal, antigen-negative cells would be stimulated in vivo by an attenuated IFNα-antibody fusion and whether the A145D mutation in IFNα is sufficient to render the fusion protein less active than a wild type IFNα fusion. Animals were treated with a 1-hr intravenous infusion of various doses of either non-targeted-IFNα(att) or non-targeted-IFNα(wt) fusion proteins. Serum levels of two known IFNα-induced biomarkers, neopterin and IP-10 [42, 43], were measured in monkey serum after each dose as indicators of systemic IFNα activity.

Non-targeted-IFNα(wt) induced substantially higher serum levels of both biomarkers compared to non-targeted-IFNα(att) (Fig 4C). For example, the levels of both neopterin and IP-10 in the monkeys given 0.1 mg/kg non-targeted-IFNα(wt) were greater than the serum levels induced in monkeys given 10 mg/kg of non-targeted-IFNα(att). This dosing difference indicates that the attenuated IFNα antibody fusion is at least 100-fold less potent in stimulating IFNα signaling on normal, antigen negative tissues than the wild type IFNα antibody fusion protein in vivo. These in vivo data confirm that the diminished off-target activity of attenuated IFNα observed in BM cells in vitro translates to a relevant primate system and suggests that the tolerable dose of an attenuated IFNα antibody fusion may likely be higher than that of wild-type IFNα immunocytokines in patients.

### Anti-CD38-IFNα(att) induces potent tumor regression and increases survival in xenograft models

**Fig 5:**
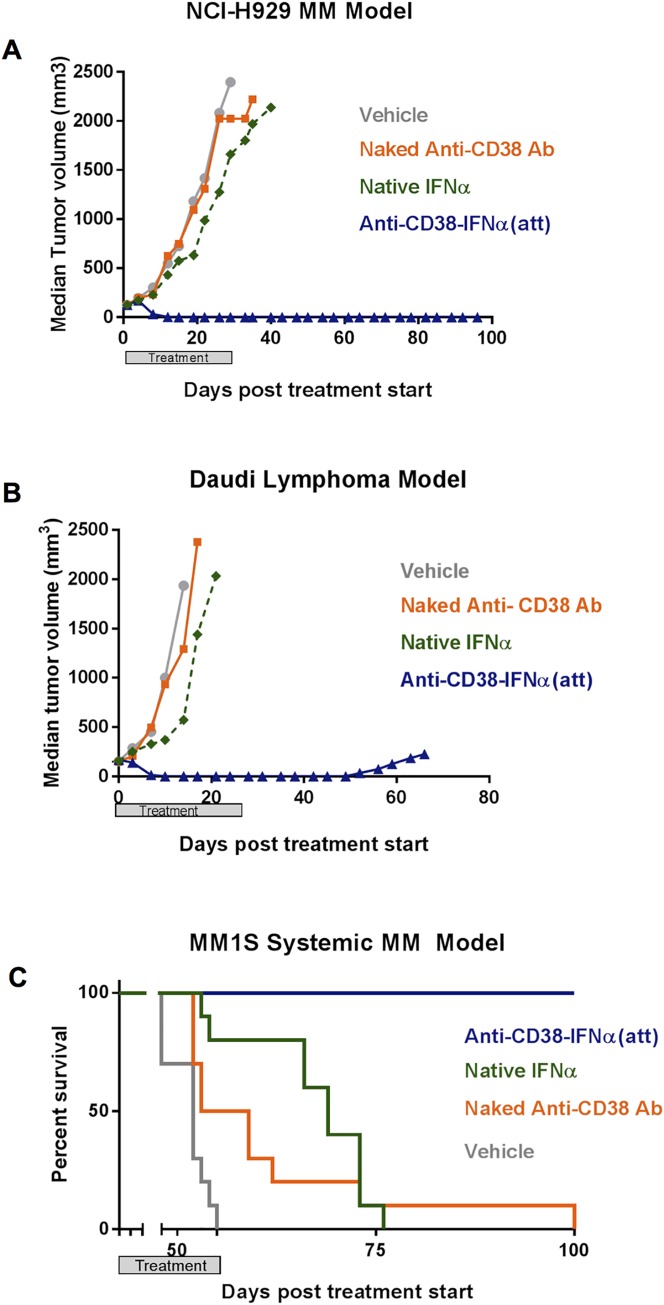
Anti-CD38-IFNα(att) induces significant, durable MM tumor regression and increases survival time in mice. (A) Inhibition of subcutaneous MM NCI-H929 tumor growth in 8–12 week old CB.17 SCID mice (n = 10 per group) that were treated twice weekly for 4 weeks with anti-CD38-IFNα(att) or naked anti-CD38 antibody (both at 10 mg/kg), native IFNα (0.4 mg/kg), or vehicle. Mice were injected subcutaneously with 1x10^7^ NCI-H929 myeloma tumor cells in 50% Matrigel and treatments began when tumors reached 120–150 mm^3^. (B) Inhibition of subcutaneous Daudi tumor growth in 6–8 week old irradiated NOD-SCID mice (n = 10 per group) treated twice weekly for 4 weeks with the same test compounds and regimen as in A. Mice were injected subcutaneously with 1 x 10^7^ Daudi Burkitt’s lymphoma tumor cells in 50% Matrigel one day after irradiation with 200rad (^60^Co). Treatments began after mean tumor size reached 169 mm^3^. (C) Percent survival of 6–8 week old irradiated NOD-SCID mice (n = 10 per group) implanted by tail vein injection of 1 x 10^7^ MM1S myeloma tumor cells. Seven days later, treatments were given twice weekly for 67 days with the same test compounds as in A. The treatment windows for all experiments are indicated by shaded bars.

To confirm that anti-CD38-IFNα(att) retains robust anti-tumor activity in vivo despite its attenuating mutation, we tested its activity in CD38 positive human MM and lymphoma xenograft tumor models. These included subcutaneous implantation models for myeloma (NCI-H929 cell line) and lymphoma (Daudi cell line), as well as a systemic model of myeloma (MM1S cell line). Treatment with anti-CD38-IFNα(att) resulted in complete regression of well-established NCI-H929 MM subcutaneous tumors (Fig 5A). Remarkably, 10 of 10 mice were tumor-free by day 22 of treatment and showed no signs of tumor regrowth for the duration of the experiment (up to 72 days after treatment cessation). In contrast, all mice treated with vehicle, anti-CD38 naked antibody, or native IFNα showed rapid NCI-H929 tumor growth, reaching tumor endpoint before day 40 (Fig 5A). Anti-CD38-IFNα(att) also had strong activity against Daudi lymphoma subcutaneous tumors relative to other treatments (Fig 5B). All Daudi tumors showed complete regression within 24 days of treatment, although small tumors did recur in 4/10 mice several weeks after treatment cessation in this model. In our studies, native IFNα treatment was ineffective in tumor cell models based on subcutaneous xenograft implants (Fig 5), whereas, native IFNα showed strong anti-tumor activity in the two LAG xenograft models which were based on in vivo passaged MM tumor fragments implanted intramuscularly (Fig 1). We postulate that IFNα was effective in the latter cases because tumor exposure to IFNα is likely much higher in tumor fragments growing in highly vascularized muscle tissue compared to cells implanted subcutaneously.

In the systemic MM model using MM1S cells, mouse body weights and overall health were monitored after the start of treatments, and survival time was the experimental endpoint. As shown in Fig 5C, all 10 mice treated with anti-CD38-IFNα(att) survived the full duration of study (>100 days) compared to a median survival time of 56 days for vehicle treated mice. Native IFNα increased median survival time by 18 days compared to vehicle (74 days vs 56 days, p = <0.001), and naked anti-CD38 antibody provided a 5 day median survival benefit over vehicle (62 days vs 56 days, p = 0.004). All statistical analyses were performed using Student’s t-Test. The results from all three xenograft models demonstrated that a strongly attenuated IFNα targeted directly to MM tumors via a CD38 antibody can induce improved, potent and long-lasting anti-tumor activity in vivo.

### Anti-CD38-IFNα(att) eliminates very large MM tumors in mice

**Fig 6:**
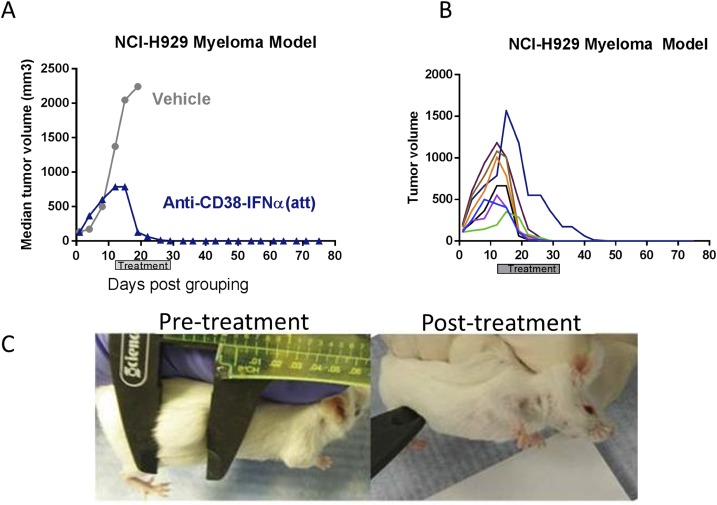
Anti-CD38-IFNα(att) induces profound anti-tumor activity on large, well-established NCI-H929 MM tumors in vivo. (A) Regression of very large (median volume = 730 mm^3^) subcutaneous NCI-H929 MM tumors in 8–12 week old CB.17 SCID mice (n = 8 per group) that were treated twice weekly for 3 weeks with 10 mg/kg of anti-CD38-IFNα(att) or vehicle. (B) Tumor volumes of the individual treated mice (n = 8) from the same experiment. (C) Images of a representative mouse from the same study with an established subcutaneous NCI-H929 tumor pre- and post-treatment with anti-CD38-IFNα(att).

The robust, and in most cases curative, anti-tumor activity observed in vivo from anti-CD38-IFNα(att) therapy prompted further evaluation of this compound in mice bearing larger tumors. We assessed its activity on NCI-H929 subcutaneous tumors that had reached an average volume of 730 mm^3^ (Fig 6). One mouse, with a tumor volume of 1800mm^3^ on day 1 of treatment, reached endpoint the following day and was excluded from the study. Of the remaining 8 treated mice, 7 were completely tumor free within 18 days of the start of treatment, and the remaining mouse was tumor free within 30 days (Fig 6A and 6B). Furthermore, no tumor regrowth was observed in any of the mice by study endpoint on day 76. These data demonstrate that targeted delivery of an attenuated IFNα molecule has profound anti-tumor activity, even on very large tumors.

### Anti-CD38-IFNα(att) provides greater anti-tumor activity than standard MM treatments

**Fig 7:**
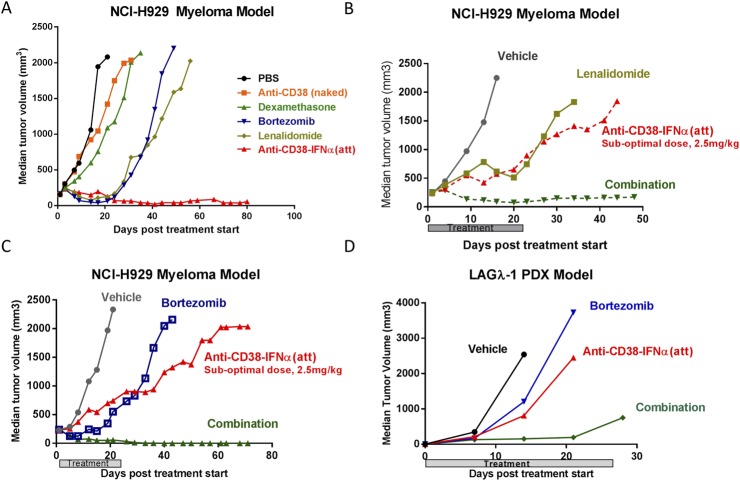
Enhanced tumor regression and synergistic activity of anti-CD38-IFNα(att) compared to standard MM therapeutics. (A) Tumor volumes from NCI-H929 tumor bearing SCID mice treated with 5mg/kg anti-CD38-IFNα(att), twice a week for 3 weeks or standard MM therapeutic agents including dexamethasone (10 mg/kg, daily for 28 days), lenalidomide (25 mg/kg, daily for 21 days), bortezomib (1 mg/kg, twice a week for 28 days), or vehicle. Average tumor size at start of treatment was 125 mm^3^. (B) NCI-H929 tumor growth inhibition and regression in SCID mice treated twice a week for 3 weeks with lenalidomide (25 mg/kg), a sub-optimal dose of anti-CD38-IFNα(att) (2.5 mg/kg), a combination of lenalidomide and anti-CD38-IFNα(att), or vehicle. Average tumor size at start of treatment was 250 mm^3^. (C) NCI-H929 tumor growth inhibition and regression in SCID mice treated twice a week for 3 weeks with bortezomib (1.0 mg/kg), a sub-optimal dose of anti-CD38-IFNα(att) (2.5 mg/kg), a combination of bortezomib and anti-CD38-IFNα(att), or vehicle. Average tumor size at start of treatment was 250 mm^3^. (D) Mouse passaged human MM xenograph LAGλ-1 tumor inhibition in SCID mice treated twice a week for 4 weeks with bortezomib (1.0 mg/kg), a high dose (10.0 mg/kg) of anti-CD38-IFNα(att), a combination of bortezomib and anti-CD38-IFNα(att), or vehicle. Treatment started on day 8 following implantation.

To assess the potency of anti-CD38-IFNα(att) compared to standard MM therapeutics, we tested anti-CD38-IFNα(att) along with several registered compounds in the NCI-H929 xenograft model. Dexamethasone, lenalidomide, and bortezomib were delivered as single agents using standard dosing regimens [44–46]. Treatment with anti-CD38-IFNα(att) generated the strongest anti-tumor response of all compounds tested in this model (Fig 7A). By day 25 of treatment, NCI-H929 tumors had completely disappeared in all mice treated with anti-CD38-IFNα(att), and all mice survived to the end of the study (day 80). In contrast, lenalidomide and bortezomib generated moderate tumor inhibition during treatments (median time to endpoint [TTE] of 55 days and 43 days, respectively), but tumors regrew in all of these animals once treatments stopped at 21 days and 28 days, respectively. Dexamethasone and the naked anti-CD38 control antibody exerted only a slight delay in NCI-H929 tumor growth compared to vehicle treatment (TTE 31 days, 26 days, and 18 days, respectively).

In follow up experiments, lenalidomide (25 mg/kg) and bortezomib (1mg/kg) were each delivered in combination with a suboptimal dose (2.5 mg/kg) of anti-CD38-IFNα(att). Both combination therapies resulted in a synergistic response with complete tumor regression in all mice within two weeks of treatment (Fig 7B and 7C) and no tumor regrowth after treatments were stopped. We also tested bortezomib plus anti-CD38-IFNα(att) combined treatment in the bortezomib-refractory LAGλ-1] MM model (Fig 7D). Because mice bearing LAGλ-1 tumors were not responsive to treatment with native IFNα (Fig 1E), we used a higher dose (10 mg/kg) of anti-CD38-IFNα(att) combined with the standard dose (1 mg/kg) of bortezomib administered twice weekly. This combination proved highly effective in reducing LAGλ-1 tumor size in all mice compared to either treatment alone, although in some mice (6/10) tumors did regrow after treatment was discontinued. Similar synergy was observed with lenalidomide plus anti-CD38-IFNα(att) combination treatment of mice bearing lenalidomide-refractory tumors (data not shown). These data suggest that even MM tumors refractory to treatment with a proteasome inhibitor can respond to combined treatment with a targeted, attenuated IFNα.

## Discussion

Our experiments confirm previous reports that IFNα has anti-myeloma activity by showing that every primary MM and MM cell line we tested was sensitive to its growth inhibitory effects at high doses. Despite strong anti-tumor activity, however, clinical use of IFNα is limited due to significant side effects at therapeutic doses. Here, we describe a novel immunological approach to minimize off-target IFNα toxicity while retaining robust on-target anti-tumor activity. This was accomplished by engineering an attenuating mutation into the IFNα portion of an immunocytokine targeted to CD38 on MM tumor cells.

Multiple groups have shown that targeting IFNα to tumor cells improves anti-tumor activity in animal studies [16, 19, 20, 24, 47]. Treatment with CD20-targeted mouse IFNα, for example, inhibited B-cell lymphoma tumor growth resulting in an 87% tumor cure rate in mice [19]. Similarly, Rossi et al. demonstrated strong anti-tumor activity of a CD20-targeted tetrameric IFNα in xenograft NHL models, and Yoo et al. showed delayed tumor growth with a CD138-targeted IFNα in a MM xenograft model [24, 47]. While these experiments convincingly demonstrate the utility of targeted IFNα immunocytokines against tumors they do not address the well documented issue of IFNα toxicity. These targeted approaches with wild type cytokines may, in fact, produce significant toxicities given the prolonged half-life of immunocytokines compared to free cytokines.

Garcin et al. recently reported use of an attenuated form of IFNα to reduce cell toxicity [21]. They fused a mutated mouse IFNα to a mLepR targeting nanobody and demonstrated strong reporter activity on target-positive HL116 cells with negligible activity on target-negative cells. Importantly, the most effective dose at inducing strong STAT1 phosphorylation in vitro had no effect on target-negative cells in vivo. We expanded upon this strategy by specifically targeting attenuated human IFNα to MM tumor cells via an anti-CD38 antibody and then tested its anti-tumor activity in vivo.

The CD38-targeted attenuated IFNα fusion protein, or “Attenukine™”, identified as anti-CD38-IFNα(att), displayed a 10,000-fold greater specificity than native IFNα for CD38-positive (tumor) vs CD38-negative (normal) cells (Fig 3C and 3D). In contrast, the corresponding wild type IFNα fusion protein showed only a 40-fold greater specificity (Fig 3A and 3B). Therefore, the attenuating mutation in the IFNα portion of the immunocytokine increased the antigen-specificity of IFNα by approximately 250-fold. This suggests that patients might safely be treated with higher doses of the anti-CD38-IFNα Attenukine™ compared to native IFNα or a conventional IFNα immunocytokine.

Other experiments reported here support a potentially favorable safety profile for an IFNα Attenukine™. Anti-CD38-IFNα(att) inhibition of normal human BM cell colony growth in vitro was ~6,000 fold weaker than native IFNα. Additionally, in a pilot cynomolgus monkey study, a non-targeted attenuated IFNα fusion protein was more than a 100-fold weaker at inducing systemic pro-inflammatory markers than the corresponding wild type IFNα fusion protein confirming that the attenuating mutation in IFNα does indeed result in lower off-target IFNα activity.

Despite strong attenuation, the CD38-targeted, attenuated IFNα provided dramatic MM anti-tumor activity in various cell line and primary-derived tumor models. In fact, anti-CD38-IFNα(att) completely eliminated established NCI-H929 MM subcutaneous tumors in all mice, and fully resolved even very large tumors. We are not aware of any other compound that has shown such profound preclinical anti-myeloma activity in vivo, especially on large established tumors. In comparison to various approved MM drugs, anti-CD38-IFNα(att) was significantly more effective in the NCI-H929 xenograft model. In addition, the strong synergistic effects of anti-CD38-IFNα(att) with both bortezomib and lenalidomide indicates that drug combinations may offer an optimal regimen in the clinic, even for bortezomib-refractory MM tumors. Finally, Daudi lymphoma tumors were highly responsive to IFNα Attenukine™ indicating that anti-CD38-IFNα(att) is likely a strong therapeutic candidate for multiple types of CD38-positive lymphoid cancers. It is important to note that while it would be most ideal to test both therapeutic activity and tolerability of IFNα and related constructs in the same animal model, this is not currently possible. Murine cells do not respond significantly to human IFNα, making mice inappropriate models for IFNα toxicity experiments. Cynomolgus monkeys serve as good animal models for tolerability to IFNα because monkey cells respond to human IFNα, but testing efficacy of drug compounds in monkeys is not possible or desirable. Thus, here we present tumor inhibition studies performed solely in mice and preliminary IFNα tolerability studies performed in monkeys.

A number of monoclonal antibodies targeting MM antigens such as CD38 and CS1(SLAMF7) have entered clinical development [48]. These antibodies lead to immune effector-mediated killing and may eliminate both normal and tumor cells expressing target antigen. Based on our results, the IgG4 anti-CD38 antibody fused to IFNα in our constructs would not be expected to kill CD38^+^ normal cells by antibody-dependent cell-mediated cytotoxicity (ADCC) or complement-dependent cytotoxicity (CDC). Rather, only IFNα-sensitive, CD38 high-expressing cells, such as MM cells, will be targeted and killed by this more selective therapeutic approach.

In summary, we describe a novel, targeted IFNα Attenukine™ that harnesses the potent anti- tumor activity of native IFNα while reducing its well documented toxic side effects. The potent anti- tumor activity with predicted lower toxicity together may provide a broader therapeutic index (TI) for the IFNα Attenukine™ compared to native IFNα or conventional IFNα immunocytokines. The remarkably durable anti-tumor activity of this new compound is unsurpassed by any current small or large molecules tested in similar MM models. Studies are underway to elucidate the possible mechanisms of action of CD38-targeted IFNα Attenukine™.
